# Feasibility of a transmucosal sublingual fentanyl tablet as a procedural pain treatment in colonoscopy patients: a prospective placebo-controlled randomized study

**DOI:** 10.1038/s41598-020-78002-0

**Published:** 2020-12-01

**Authors:** Mari Fihlman, E. Karru, P. Varpe, H. Huhtinen, N. Hagelberg, T. I. Saari, K. T. Olkkola

**Affiliations:** 1 Department of Anaesthesiology and Intensive Care, University of Turku, Kiinamyllynkatu 4-8, P.O. Box 52, 20520 Turku, Finland; 2 Division of Perioperative Services, Intensive Care and Pain Medicine, Turku University Hospital, Turku, Finland; 3 Department of Digestive Surgery, Turku University Hospital, Turku, Finland; 4 Pain Clinic, Turku University Hospital, Turku, Finland; 5 Department of Anaesthesiology, Intensive Care, and Pain Medicine, University of Helsinki and HUS Helsinki University Hospital, Helsinki, Finland

**Keywords:** Gastroenterology, Medical research, Pain

## Abstract

Since patients often experience pain and unpleasantness during a colonoscopy, the present study aimed to evaluate the efficacy and safety of sublingually administered fentanyl tablets for pain treatment. Furthermore, since the use of intravenous drugs significantly increases colonoscopy costs, sublingual tablets could be a cost-effective alternative to intravenous sedation. We conducted a prospective placebo-controlled randomized study of 158 patients to evaluate the analgesic effect of a 100 µg dose of sublingual fentanyl administered before a colonoscopy. Pain, sedation, nausea, and satisfaction were assessed during the colonoscopy by the patients as well as the endoscopists and nurses. Respiratory rate and peripheral arteriolar oxygen saturation were monitored throughout the procedure. There were no differences between the fentanyl and placebo groups in any of the measured variables. The median pain intensity values, as measured using a numerical rating scale, were 4.5 in the fentanyl group and 5 in the placebo group. The sedation and oxygen saturation levels and the respiratory rate did not differ between the groups. The majority of the colonoscopies were completed.Our results indicate that a 100 µg dose of sublingual fentanyl is not beneficial compared to the placebo in the treatment of procedural pain during a colonoscopy.

## Introduction

A colonoscopy is an invasive procedure that can cause pain and discomfort in patients. Various drugs are used to prevent and treat discomfort during a colonoscopy, but the optimal drugs and dosing regimens are still unknown. Patients want to have a painless colonoscopy^1^, which can be achieved with analgesics and/or sedatives. Even though a colonoscopy can be successfully performed with drugs that do not alter the patient’s level of consciousness or cause nociception^2–4^, the use of intravenous sedation or analgesia has become the standard practice in many countries^5,6^.

Most patients find procedural sedation effective, but it may increase the risks and costs of the procedure^7,8^. Repeated dosing of sedatives can cause prolonged sedation, hypoxia, and respiratory depression by decreasing the patient’s respiratory response to carbon dioxide^9^. Since oxygenation monitoring is required in such cases, patient follow-up is essential, and intravenously sedated patients cannot be discharged immediately after the colonoscopy.

Typically, intravenous midazolam is used to induce sedation for a colonoscopy. However, it has been shown that low-dose midazolam neither relieves discomfort nor produces amnesia in patients^10^. A recent meta-analysis suggested that propofol is superior to other sedative agents; recovery and discharge times were shorter and patient satisfaction scores were greater than with benzodiazepines^11^. However, sedation with propofol is associated with longer recovery times than sedation with midazolam and fentanyl, especially in the elderly^12^. Meanwhile, previous studies have interestingly demonstrated that patient comfort appears to be unrelated to the use of sedatives or analgesics^10,13^. Obviously, further studies are needed on procedural sedation during colonoscopy.

Although many patients report pain during a colonoscopy, only a few studies have focused on the use of opioid analgesics during this procedure^14–16^. Fentanyl is rather short acting compared to other strong opioids (e.g., sufentanil) that are commonly used during the intraoperative period. Transmucosal sublingual fentanyl has been developed to improve the management of breakthrough pain in cancer patients. Achieving analgesia using a transmucosal tablet during colonoscopy is a novel approach, and a recent study reported promising results that indicated that oral transmucosal fentanyl is a safe and effective premedication in patients undergoing surgery under general anesthesia^17^. Similarly, low doses of intravenous fentanyl seem to be effective in achieving a satisfactory level of comfort during a colonoscopy^14^. Sublingual administration is an easy and non-invasive method of fentanyl administration prior to a colonoscopy. Furthermore, a single dose of sublingual fentanyl does not require follow-up after the procedure. The only limitation is that the patient is not allowed to drive a vehicle after the administration of strong opioids.

The purpose of the present study was to compare the efficacy and safety of sublingually administered fentanyl and a placebo in patients undergoing a colonoscopy. We hypothesized that sublingual fentanyl would provide sufficient analgesia and increase patient satisfaction during the colonoscopy.

## Methods

### Study design

In this randomized, double-blind, and placebo-controlled study, 158 patients undergoing a diagnostic or therapeutic colonoscopy were randomly given 100 µg dose of sublingual fentanyl (Abstral 100 µg, ProStrakan) or the placebo before the procedure. The dose was based on a small pilot study of 35 patients prior to the initiation of the current study. In the pilot study, a 100 µg dose of sublingual fentanyl appeared to be a useful alternative to analgesia and sedation during an endoscopic procedure. The patients were randomized into two groups using the sealed envelope technique, and all patients, investigators, and staff members involved in the study were blinded to the treatment assignment.

### Ethical considerations

This study was approved by the institutional review board (IRB) of the Hospital District of Southwest Finland (IRB number: 140/2011). The trial was registered before patient enrollment at clinicaltrials.gov (NCT01604187, Principal investigator: M.F., Date of registration: 22/05/2012) and in the EudraCT database (2011-005688-26, Principal investigator: M.F., Date of registration: 21/11/2011). All research was performed in accordance with relevant guidelines and regulations. Written informed consent was obtained from each patient.

### Subject eligibility

Male and female patients between 18 and 80 years old who were scheduled for a routine diagnostic or therapeutic colonoscopy and had an American Society of Anesthesiologists (ASA) physical status classification of I–III, a body mass index (BMI) < 35, and a weight of more than 50 kg were deemed eligible for the present study. Patients who had a previous gastrointestinal surgery, sleep apnea, chronic obstructive pulmonary disease, or SpO_2_ < 90%, who were pregnant or nursing, or who were undergoing concomitant drug therapy known to cause significant enzyme induction or CYP3A4 inhibition were excluded from the study. Additional exclusion criteria were a history of intolerance to fentanyl or related compounds; a history of or current alcoholism or drug abuse; and a history of and psychiatric, psychological, or other emotional problems that were likely to invalidate informed consent.

### Study procedures

Consecutive ambulatory colonoscopy patients were randomized to receive a sublingual fentanyl tablet (Abstral 100 µg, ProStrakan) or a matching placebo tablet 10 min before the procedure. The patients were instructed not to swallow the tablet but to allow it to completely dissolve in the sublingual cavity without chewing or sucking. The patients were not allowed to eat or drink anything until the sublingual tablet was completely dissolved. The patients did not receive any other sedative or analgesic drug during the colonoscopy.

The patients assessed their average pain intensity using a numerical rating scale (NRS 0–10) during and at the end of the colonoscopy. Adverse opioid effects and subjective effects were recorded at the end of the colonoscopy. The following adverse opioid effects were evaluated using NRS (0–10): drowsiness (alert/very drowsy), pleasantness (very unpleasant/very pleasant feeling), and nausea/vomiting (no nausea/very strong nausea). At the end of the colonoscopy, the endoscopists and the assisting nurses used NRS to evaluate whether the patient seemed to have pain or nausea and judged the level of sedation and the overall flow of the procedure. The endoscopists also estimated any technical difficulties associated with the colonoscopy. SpO_2_ and the respiratory rate were followed throughout the procedure. If SpO_2_ decreased below 90% or the respiratory rate decreased below eight per minute, additional oxygen was given. In the case of excessive opioid effects, 0.1 mg of intravenous naloxone was given.

The patients scored their overall satisfaction with the procedure prior to discharge. In addition, they were interviewed by telephone one day after the procedure. At this time, they assessed their anxiety before the procedure using a scale from 1 to 4 (1 = no anxiety, 4 = maximal anxiety) and evaluated how well they could remember events during and after the colonoscopy using a scale from 1 to 4 (1 = remember everything, 4 = cannot remember anything). Their level of abdominal pain was assessed using a scale from 1 to 4 (1 = no pain, 4 = a lot of pain). The patients were also asked whether they had any adverse effects during the day after the procedure, such as abnormal tiredness, nausea, or dizziness; they evaluated these effects using a scale from 1 to 4 (1 = no drowsiness/nausea/dizziness, 4 = a large amount of drowsiness/nausea/dizziness). Finally, patients assessed the unpleasantness of the colonoscopy from 1 to 4 (1 = not at all, 4 = very unpleasant).

### Statistics

Based on previous study^18^, it was calculated that 87 patients would be needed per group to demonstrate a 30% decrease in the worst experienced pain at a level of significance 0.05 and power of 90%. The standard deviation of the worst experienced pain was assumed to be 50% of the mean pain score in the placebo group. Because of possible dropouts, 100 patients were planned to be recruited to both groups. However, because it was unexpectedly difficult to recruit suitable patients, we decided to settle for a smaller power. We estimated that we needed 73 patients each in the fentanyl and placebo groups to demonstrate, using NRS, a 25% difference in the worst experienced pain at a significance level of 0.05 and a power of 85%. Because of possible dropouts, 75 patients were planned to be recruited to both groups. The primary efficacy analysis was summarized descriptively for overall success evaluated by the patients, endoscopists and nurses and by treatment group. We performed the chi-square test or the Fisher’s exact test where appropriate and used the analysis of variance model with treatment as the main effect for overall and pairwise comparisons.

## Results

A total of 158 patients at Turku University Hospital were recruited between April 2012 and December 2018. Fourteen patients were excluded from the study because of missing data. As such, there were 72 patients in each group. Descriptive data are summarized in Table 1.

**Table 1:** Clinical characteristics of the studied patients.

	Placebo (n = 72)	Fentanyl (n = 72)
Mean age, years (SD)	59.9 (13.5)	57.9 (13.6)
Male/female, n	39/33	39/33
Mean height, cm (SD)	170 (9.7)	172 (8.9)
Mean weight, kg (SD)	79.3 (14.8)	82.9 (19.5)
Median lenght of the procedure, min (range)	15 (5–40)	20 (5–50)
Median time between the end of colonoscopy and hospital checkout, min (range)	25 (5–90)	25 (9–60)

There were no differences in pain intensity or degree of sedation between the fentanyl and placebo groups. The maximum pain experienced by patients was 7 [4.75–8] in the fentanyl group and 8^5–9^ in the placebo group, respectively. The fentanyl group had a median pain intensity value of 4.5, and the placebo group had a median pain intensity value of 5. The female patients had a median pain intensity value of 5 in both groups, and the male patients of 3 and 4 in the fentanyl and placebo groups, respectively. There was no statistical difference between the groups in male and female patients (*p* = 0.124). Both groups had a median self-reported degree of sedation of 0. The results are summarized in Table 2.

**Table 2:** Assessment of subjective parameters by patient (n = 144), surgeon and nurse using numerical rating scale (0–10). Data are shown as median and interquartile range.

Parameter	Patient			Surgeon			Nurse			
	Fentanyl	Placebo	p-value	Fentanyl	Placebo	p-value	Fentanyl	Placebo	p-value	
Progression of procedure	NA	NA	–	9 [8–10]	9 [8–10]	0.992	8 [7–9]	9 [7–9]	0.834	
Co-operation	NA	NA	–	10 [9.75–10]	10 [9.75–10]	0.132	10 [9–10]	10 [9–10]	0.752	
Average pain	4.5 [2–7]	5 [3–6.5]	0.852	5 [2–7]	5.5 [3–8]	0.132	6 [2–7]	6 [3–8]	0.716	
Maximum pain	7 [4.75–8]	8 [5–9]	0.212	NA	NA	–	NA	NA	–	
Sedation	NA	NA	–	0 [0–1]	0 [0–1]	0.910	1 [0–2]	0 [0–1]	0.093	
Nausea	0 [0–1]	0 [0–0]	0.255	0 [0–0]	0 [0–0]	0.568	0 [0–0]	0 [0–0]	0.603	
Unpleasantness	4 [2–6]	5 [3–7]	0.369	NA	NA	–	NA	NA	–	
Drowsiness	0 [0–0]	0 [0–0]	0.595	NA	NA	–	NA	NA	–	

Three procedures (one in the fentanyl group and two in the placebo group) were interrupted due to excessive pain. Both groups had a median time interval between the end of the colonoscopy and hospital discharge of 25 min (fentanyl group: range = 9–60 min, placebo group: range = 5–90 min) (Table 1).

Oxygen saturation was in the normal range in both groups, and there were no desaturation events. Respiratory rates were above eight per minute, and naloxone was not needed in any of the patients. Post-procedural interviews the day after the colonoscopy showed no differences in patient experience reminiscence between the fentanyl and placebo groups (Table 3).

**Table 3:** Post procedure interview by telephone on the first day after the procedure. Assessment of subjective parameters by patient using numerical rating scale (0–4). Data are shown as median and interquartile range.

	Placebo (n = 72)	Fentanyl (n = 72)
Anxiety before the procedure	2 [2–3]	2 [2–3]
Recall during the procedure	1 [1–1]	1 [1–1]
Recall after the procedure	1 [1–1]	1 [1–1]
Stomach pain	1 [1–2]	1 [1–2]
Nausea	1 [1–1]	1 [1–1]
Dizziness	1 [1–2]	1 [1–2]
Drowsiness	1 [1–2]	1.5 [1–2]
Unpleasantness	2 [2–3]	2 [2–3]

## Discussion

The study findings indicate that a 100 µg dose of sublingual fentanyl did not affect colonoscopy-related pain intensity compared to the placebo. The sublingual fentanyl did not cause significant adverse effects or sedation, and patient hospital stays were not prolonged. Although patients reported moderate pain, nearly all of the procedures were completed successfully. The fentanyl did not have a significant effect on the satisfaction of the endoscopists. Finally, there were no differences between the fentanyl and placebo groups on the day after the procedure when the patients were asked to assess their anxiety level before the procedure.

The primary aim of this study was to evaluate the efficacy of a single sublingual dose of fentanyl in relieving pain during a colonoscopy. Sublingual administration was chosen to avoid the need for intravenous access. Although Singh et al. showed that a 200 µg dose of transmucosal fentanyl as a premedication in surgical patients undergoing general anesthesia caused effective anxiolysis with minimal adverse effects^17^, we found no difference between the fentanyl and placebo groups when pain during the colonoscopy was assessed using NRS. Similarly, a previous study evaluating the effect of a 200 µg dose of sublingual fentanyl as a premedication before bone marrow aspiration and biopsy^19^ found the fentanyl to be inadequate in relieving patients’ pain.

In the present study, the median NRS pain values were 4.5 in the fentanyl group and 5 in the placebo group, which is commonly considered moderate pain. In a study by Lazaraki et al., patients received a small dose of intravenous fentanyl or midazolam, and the mean pain scores (on a scale of 0–10) were 2.59 in the fentanyl group and 4.43 in the midazolam group^14^. Meanwhile, when the effects of administering 1,000 mg of intravenous paracetamol and 0.5–1 µg/kg of intravenous fentanyl to colonoscopy patients were compared, the mean pain assessments were 4.00 and 3.77, respectively^20^. Thus, in our study, the median pain scores were similar to previous findings.

The bioavailability of sublingual fentanyl is approximately 70%, and a 100 µg dose of sublingual fentanyl—as used in the present study—has been shown to be effective and safe in average-sized adults and in elderly patients with comorbidities^21–23^. Regarding the intravenous route, a single 36 µg dose of intravenous fentanyl has been shown to be sufficient prior to colonoscopy^14^. In the pilot study, the 100 µg dose of sublingual fentanyl did not delay home readiness following the endoscopic procedure. For safety and ethical reasons, we wanted to keep the dose of fentanyl relatively low, especially since most patients were opioid naïve. Furthermore, although, according to its summary of product characteristics, Abstral is contraindicated for opioid intolerant patients and the treatment of acute pain, we considered its off-label use in procedural sedation under the supervision of a senior anesthesiologist trained to use fentanyl to be acceptable for the present study purposes.

Our results indicate that a single sublingual low-dose opioid is not an ideal premedication for patients having a colonoscopy. Furthermore, we hypothesize that pain is seldom the only limiting factor for success in a colonoscopy; discomfort and anxiety might also be of importance. Although pain is a subjective experience, we evaluated analgesia by recording self-reported pain ratings using NRS, which is a reliable and validated method for quantifying pain^24^. The patients in either group did not feel sedated, indicating that pain is probably the worst part of a colonoscopy and that pain, anxiety, and discomfort should be treated together.

Moderate or conscious sedation is safer and more cost efficient than deep sedation^25^. However, since there are very few studies in which analgesics alone have been used to treat pain during a colonoscopy, comparing sedation levels to those in other studies is difficult. Most studies used a combination of an opioid and midazolam to achieve sedation^26,27^. In the present study, the endoscopists and the assisting nurses assessed the patients’ sedation levels using NRS and determined the median level of sedation to be 0 in both groups. The median time between the end of the colonoscopy and hospital checkout was very short in both groups: 25 (range 5–90) minutes in the placebo group and 25 (range 9–60) minutes in the fentanyl group. To ensure the smooth operation of a colonoscopy unit, it is important that patients recover rapidly from sedation. The time required to reach home discharge readiness was found to be lower in patients who received only intravenous fentanyl for sedation than those who received only midazolam^14^. Routinely given conscious sedation does not benefit patients or make a colonoscopy technically easier^4^, and premedication or sedation should only be offered to very anxious patients.

Our study has limitations, one of which is the long recruitment period. The main reasons for this were patients’ unwillingness to undergo a colonoscopy without sedation and the proscription of driving a car after the administration of the study drugs. Another limitation was the timing of the fentanyl dose. Patients received the drug 10 min before the procedure, which coincides with the first detectable concentrations and reported onset of effect^28^. However, the peak concentration of sublingual fentanyl in the blood was reached 39.7 min after the 100 µg dose of sublingual fentanyl was administered. Since the median length of the colonoscopy in the current study was short (15 min in the placebo group and 20 min in the fentanyl group), the patients could have had better pain relief during the procedure if the time interval between the administration of the drug and the beginning of colonoscopy had been longer. Meanwhile, although the 100 µg dose of sublingual fentanyl was considered adequate based on previous reports^14^, the lack of difference in the efficacy of the fentanyl and the placebo is a strong indication that the dose of fentanyl was probably insufficient.

In conclusion, a single 100 µg dose of sublingual fentanyl as a monotherapy administered 10 min before the start of an endoscopic procedure was not found to be beneficial compared to the placebo in the treatment of procedural pain during a colonoscopy. Current ambulatory practice requires fast patient turnover, an efficient and short-acting analgesic, and the rapid discharge of patients. It remains to be elucidated whether a combination of fentanyl and oral benzodiazepine would be more effective, equally safe, and able to eliminate the need for deeper intravenous sedation during a colonoscopy.
